# Comparing the speed of irrigation between pulsatile lavage versus gravity irrigation: an Ex-vivo experimental investigation

**DOI:** 10.1186/s13037-017-0124-2

**Published:** 2017-03-27

**Authors:** Lily R. Mundy, Mark J. Gage, Richard S. Yoon, Frank A. Liporace

**Affiliations:** 1 Division of Plastic and Reconstructive Surgery, Duke University, Durham, NC USA; 2 Section of Orthopaedic Trauma, Department of Orthopaedic Surgery, Duke Univsersity, Durham, NC USA; 3 Division of Orthopaedic Trauma, Department of Orthopaedic Surgery, Jersey City Medical Center – RWJBarnabas Health, 355 Grand Ave, Jersey City, NJ 07302 USA

**Keywords:** Irrigation and debridement, Pulsatile lavage, Gravity lavage, Open fracture, Pulse lavage

## Abstract

**Background:**

The need for reoperation or wound infection treatments between pulsatile and gravity irrigation are statistically equivalent, however, it is unclear which method maximizes operative efficiency and expeditious irrigation. In this study we set out to determine the differences in irrigation rate between these various treatment methods.

**Methods:**

This was an ex-vivo experimental laboratory study not involving human subjects. Irrigation rates were tested based on the time in seconds required to empty a three-liter bag of normal saline hanging at either 6 or 9 ft. Three forms of irrigation were tested: gravity irrigation (GI6, GI9), low-pressure pulsatile irrigation (LP6, LP9) and high-pressure pulsatile irrigation. One-way ANOVA and Student’s *t*-test were used to compare rates based on height and form of irrigation.

**Results:**

Significant differences in irrigation rates were noted at 6 ft between all three forms of irrigation with gravity irrigation the fastest followed by high-pressure and low-pressure pulsatile irrigation (GI6, mean 142 s ± 3.2; HP6, mean 189 s ± 10.2; LP6, mean 323 s ± 22.5; *p* < 0.001). This difference was also found at 9 ft (GI9, mean 114 s ± 1.5; HP9, mean 186 s ± 10.5; LP9, mean 347 s ± 3.5; *p* < 0.001). Gravity irrigation was significantly faster (*p* < 0.001) at an increased height, whereas the high and low-pressure irrigation rates were unaffected by height. List price comparison found pulsatile irrigation to cost approximately 3.3 times more than gravity lavage.

**Conclusions:**

Gravity irrigation provided the most rapid rate of irrigation tested, regardless of the height. With existing literature demonstrating equivalent clinical outcomes between methods, gravity lavage offers a faster and potentially more cost-effective form of irrigation.

## Background

Along with immediate antibiotic administration, thorough debridement and irrigation is a critical step in the management of infection prevention in patients with open fractures. Debridement and irrigation creates a clean healing base by decreasing bacteria load, removing foreign bodies and debrided necrotic tissue [1–3]. While the utility of debridement is well established, controversy lies in the specific type of irrigation that should be utilized [4–9].

The most commonly utilized techniques in the irrigation of open fractures are gravity irrigation and pulse irrigation. These techniques differ in two main ways: the presence of pulsation and the maximum force applied to the tissues. Pulse irrigation can generate low (5–10 pounds per square inch (psi)) or high (20+ psi) pressure pulsations, whereas gravity irrigation results in very low continuous pressures (1–2 psi) [10].

Numerous studies have attempted to demonstrate the superiority of one method or pressure gradient, however there are conflicting results. High-pressure irrigation has traditionally been thought to be more effective at removing bacteria from tissues [1, 11, 12]. However, it has also been associated with a greater degree of damage to bone and soft tissue and thought to potentially drive bacteria deeper into tissues [11, 13–18]. Recently, results of the Fluid Lavage in Open Wounds (FLOW) trial have helped to establish that there are no significant differences in the need for reoperation or wound infection treatments between high or low-pressure pulse irrigation or gravity irrigation [10]. However, it is unclear which method is fastest, resulting in the most expeditious irrigation. In this study, we set out to determine the differences in irrigation rate between these various treatment methods.

## Methods

This was an ex-vivo experimental laboratory study not involving human subjects. Institutional review board (IRB) was not required in the completion of this study. Irrigation rates were tested based on the time in seconds required to empty a three-liter bag of normal saline, completed by the authors. Each form of irrigation was tested with saline bags hanging at either 6 ft or 9 ft off the ground.

Three forms of irrigation were tested at each height:

- Gravity Irrigation (GI6, GI9)
- Pulsatile Irrigation at a low-pressure setting (LP6, LP9)
- Pulsatile irrigation at a high-pressure setting (HP6, HP9).

The exiting height of either the gravity irrigation tubing or the pulsatile lavage was held at 3 ft above the ground and 5 ft away from the bag of irrigation to simulate a common positioning in the operating room. The gravity irrigation had a total tubing length of 94 in. and the pulse irrigator tubing was 108 in. in length. Utilizing a digital timer, total time was measured (in seconds) by a research assistant. Total time recorded was from the point when saline started to flow from the tip of the gravity tubing or the pulse lavage until no further fluid was being released. Three flow cycles were completed and timed for each specific irrigation height and flow combination.

One-way ANOVA and Student’s *t*-test was used to compare irrigation rates based on height and form of irrigation. Significance was set to *p* < 0.05.

## Results

At irrigation height of 6 ft, significant differences in irrigation rates were noted between all three forms of irrigation (Table 1). Gravity irrigation reported the fastest times followed by high-pressure pulsatile irrigation and low-pressure irrigation (GI6, mean 142 s ± 3.2; HP6, mean 189 s ± 10.2; LP6, mean 323 s ± 22.5; *p* < 0.001, Table 1). The same significant difference was also found at 9 ft (GI9, mean 114 s ± 1.5; HP9, mean 186 s ± 10.5; LP9, mean 347 s ± 3.5; *p* < 0.001, Table 1).

**Table 1:** Total mean time values, standard deviations (SD) with ANOVA statistical comparisons

	Gravity Irrigation (sec)	Pulsatile (Low)	Pulsatile (High)	*p* value (ANOVA)
6 ft, mean (sd)	141.7 (3.2)	323.7(22.5)	189.0(10.1)	*p* < 0.001
9 ft, mean (sd)	114.3 (1.5)	347(3.5)	186(10.5)	*p* < 0.001

*Sd* standard deviation, *ft* feet, *sec* seconds, *ANOVA* analysis of variance

When comparing irrigation types at the two different heights, head-to-head comparisons noted that gravity irrigation was significantly faster (*p* < 0.001) at the increased height whereas the high- and low-pressure irrigation rates were unaffected by height (Table 2). Finally, utilizing publically available pricing, gross cost comparison found pulsatile irrigation to cost approximately 3.3 times more than the gravity lavage tubing used in the study.

**Table 2:** Head-to-Head Comparisons between gravity irrigation (GI), low pulsatiles (LP) and high pulsatile (HP) values, statistical comparision via Student’s *t*-test

Head-to-Head Comparison	*p* value (*t*-test)
GI6 vs. GI9	*p* < 0.001
LP6 vs. LP9	*p* = 0.15
HP9 vs. HP9	*p* = 0.74
GI6 vs, LP6	*p* < 0.001
GI6 vs. HP6	*p* = 0.002
GI9 vs.LP9	*p* < 0.0001
GI9 vs. HP9	*p* = 0.0003
LP6 vs. HP6	*p* = 0.0006
LP9 vs. HP9	*p* < 0.0001

*GI6* gravity irrigation at 6 ft, *GI9* gravity irrigation at 9 ft, *LP6* low-pressure pulse lavage at 6 ft, *LP9* low-pressure pulse lavage at 9 ft, *HP6* high-pressure pulse lavage at 6 ft, *HP9* high-pressure pulse lavage at 9 ft

## Discussion

In evaluating irrigation techniques, the primary variables are volume of irrigant, additives to the irrigation, the pressure applied to tissues, and the method of irrigant delivery. All of these variables have been studied in detail, with the goal of identifying the ideal irrigation technique. One of the least debated findings is that greater volumes of irrigation have been associated with greater reductions in bacteria load [9, 13, 14]. The quantity of irrigation to optimize outcomes has not been established. However, a review by Anglen et al. gives the following suggestion: 3 liters for Grade I fractures, 6 liters for Grade II fractures, and 9 liters for Grade III fractures [13]. Alternatively, the FLOW investigators suggest 3 liters for Grade I fractures and 6 liters for Grade II and III fractures [10]. Thus, our results are listed in irrigation time per 3-liter quantities of fluid.

Additionally, multiple studies have shown no added value in irrigation additives such as soap or antibiotics in comparison to normal saline [7, 8, 13, 17]. Furthermore, soap has frequently been shown to have worse outcomes in comparison to normal saline in some studies, especially noted in the recent FLOW trial [10].

As previously discussed, prior to the recent study published by the FLOW investigators [10], there has been significant controversy in the most ideal pressure settings for irrigation. Many of the previous studies regarding both irrigation pressures and irrigant additives had endpoints that included either tissue culture data or bioluminescent patterns of luminescent bacterium that had inoculated the wound. While instructive from a basic science standpoint, the majority of these studies failed to provide clinically relevant endpoints to change a surgeon’s practice habits. In the FLOW study, however, the endpoints were all clinically driven, with outcomes measured based on re-operation rates. The authors found no difference in outcomes between very low, low, and high-pressure irrigation in open fractures. Thus, this study answered an important clinical question regarding irrigation of open fractures: within commonly utilized irrigation techniques, pressure does not matter.

Given the clinical equivalence between irrigation methods, the relative speed of irrigation becomes important. A study by Karuppasamy et al. in 2004 [19] attempted to answer this question. The authors compared rates of irrigation between pulse irrigation, syringe irrigation with a 19-gauge needle, standard IV tubing with or without a pressure bag, and their technique of cutting off the nozzle of a saline bag and manually squeezing the bag. The manual technique of squeezing the saline bag without any associated tubing was found to be the fastest, taking 30 s to empty 1 liter of saline.

Limitations to this manual technique were highlighted in a response by Joshi et al. [20] and included the need for the irrigator to use both hands, thus limiting one’s ability to provide exposure or suction while irrigating. Furthermore, all of the compared irrigation methods in this paper, with the exception of pulse irrigation, are not frequently utilized in the irrigation of open fractures. Thus, the goal of our study was to identify the most expeditious method of irrigation, using the most common methods of open fracture irrigation: gravity irrigation with cystoscopy tubing and pulse irrigation.

Our comparison demonstrated that gravity irrigation was the fastest method of irrigation, with faster times associated with higher saline bag heights. Pulse irrigation, despite having a motor controlled device, was slower. The smaller diameter of the irrigation outlet in the pulse irrigator, a property that increases flow pressure, was likely one of the driving factors resulting in a significantly slower flow of water in comparison to gravity irrigation. Additionally, gravity irrigation was faster at a higher height. This is likely the result of a more direct path of tubing, along with a more vertical path of flow, both decreasing the friction and turbulent flow associated with redundancy in tubing and resulting in an increased velocity. Lastly, gross cost analysis noted a 3.3 times higher cost for pulsatile devices; albeit, depending on pricing at individual institutions, this may or may not be applicable. Therefore, in combination with results of the FLOW study, gravity irrigation was previously demonstrated to be clinically equivalent to pulse irrigation, and has now been demonstrated to be both faster and cheaper, making it the most expeditious option, both clinically and monetarily.

The strengths of our study are as follows. We are able to provide surgeons with a clear answer regarding the fastest irrigation method, associated with the volume and methods of irrigation that are most frequently utilized. In addition, we have demonstrated changes in irrigation rates associated with the height of the bag. Furthermore, we determined this in a simple and unbiased way. These results are not subject to an individual’s strength in manually applying pressure to the irrigation, or the variability in time associated with either hanging or providing the surgeon with multiple bags of irrigation.

There are limitations to our study. This study was performed in an artificial environment. Irrigation times were measured without a patient on the operating table irrigating an actual wound. The irrigation solution was simply allowed to empty out in an environment that attempted to stimulate the operating room, with the saline hung at the head of the bed and the tubing draped over the operating table. However, these factors were held constant across the irrigation methods, mitigating the impact on our findings. In addition, the brands, and thus outflow diameters, as well as the assembly and use of cysto tubing and pulse irrigators vary across hospitals. A key component in the rate of fluid flow is the diameter of the tubing, thus results may vary across different hospitals. Lastly, the utility of our study relies on previously published data. If there are differences in outcomes related to irrigation technique, then our results effectively become irrelevant.

## Conclusions

In summary, the fastest and most cost effective method is gravity irrigation. Furthermore, placing the gravity irrigation saline bag at an increased height results in increased flow and decreased irrigation time. It is important to note that debridement still remains the mainstay in the treatment of any open fracture or wound, and futures studies may or may not show the importance of irrigating at all. However, until future studies provide further insight, gravity irrigation at a height above 6 ft is the most expeditious mode of saline delivery.

## Abbreviations

GI6: Gravity irrigation 6 ft
GI9: Gravity irrigation 9 ft
HP6: High pressure pulsatile 6 ft
HP9: High pressure pulsatile 9 ft
IRB: Institutional review board
LP6: Low pressure pulsatile 6 ft
LP9: Low pressure pulsatile 9 ft
